# The Catalan Surveillance Network of SARS-CoV-2 in Sewage: design, implementation, and performance

**DOI:** 10.1038/s41598-022-20957-3

**Published:** 2022-10-06

**Authors:** Laura Guerrero-Latorre, Neus Collado, Nerea Abasolo, Gabriel Anzaldi, Sílvia Bofill-Mas, Albert Bosch, Lluís Bosch, Sílvia Busquets, Antoni Caimari, Núria Canela, Albert Carcereny, Carme Chacón, Pilar Ciruela, Irene Corbella, Xavier Domingo, Xavier Escoté, Yaimara Espiñeira, Eva Forés, Isabel Gandullo-Sarró, David Garcia-Pedemonte, Rosina Girones, Susana Guix, Ayalkibet Hundesa, Marta Itarte, Roger Mariné-Casadó, Anna Martínez, Sandra Martínez-Puchol, Anna Mas-Capdevila, Cristina Mejías-Molina, Marc Moliner i Rafa, Antoni Munné, Rosa Maria Pintó, Josep Pueyo-Ros, Jordi Robusté-Cartró, Marta Rusiñol, Robert Sanfeliu, Joan Teichenné, Helena Torrell, Lluís Corominas, Carles M. Borrego

**Affiliations:** 1 Catalan Institute for Water Research (ICRA), Emili Grahit 101, 17003 Girona, Catalonia Spain; 2 Universitat de Girona, 17003 Girona, Catalonia Spain; 3 Centre for Omic Sciences (COS), Joint Unit Universitat Rovira i Virgili-EURECAT, Unique Scientific and Technical Infrastructures (ICTS), Eurecat, Centre Tecnològic de Catalunya, Avinguda Universitat 1, 43204 Reus, Catalonia Spain; 4 Applied Artificial Intelligence Technological Unit, Eurecat, Centre Tecnològic de Catalunya, Science and Technology Park, H3, 25003 Lleida, Catalonia Spain; 5 Laboratory of Viruses Contaminants of Water and Food, Department of Genetics, Microbiology and Statistics, School of Biology, University of Barcelona, 08028 Barcelona, Catalonia Spain; 6 Enteric Virus Laboratory, Section of Microbiology, Virology and Biotechnology, Department of Genetics, Microbiology and Statistics, School of Biology, University of Barcelona, 08028 Barcelona, Catalonia Spain; 7 Research Institute of Nutrition and Food Safety (INSA), University of Barcelona, 08921 Santa Coloma de Gramenet, Catalonia Spain; 8 Biotechnology Area, Eurecat, Centre Tecnològic de Catalunya, Avinguda Universitat 1, 43204 Reus, Catalonia Spain; 9 Public Health Agency of Catalonia (ASPCAT), Roc Boronat 81-95, 08005 Barcelona, Catalonia Spain; 10 Technological Unit of Nutrition and Health, Eurecat, Centre Tecnològic de Catalunya, Avinguda Universitat 1, 43204 Reus, Catalonia Spain; 11 Catalan Water Agency (ACA), Provença, 260, 08008 Barcelona, Catalonia Spain; 12 Institute of Environmental Assessment & Water Research (IDAEA), CSIC, Barcelona, Catalonia Spain; 13 Group of Molecular Microbial Ecology, Institute of Aquatic Ecology, University of Girona, 17003 Girona, Catalonia Spain

**Keywords:** Environmental microbiology, Virology, Microbiology, Environmental sciences

## Abstract

Wastewater-based epidemiology has shown to be an efficient tool to track the circulation of SARS-CoV-2 in communities assisted by wastewater treatment plants (WWTPs). The challenge comes when this approach is employed to help Health authorities in their decision-making. Here, we describe the roadmap for the design and deployment of SARSAIGUA, the Catalan Surveillance Network of SARS-CoV-2 in Sewage. The network monitors, weekly or biweekly, 56 WWTPs evenly distributed across the territory and serving 6 M inhabitants (80% of the Catalan population). Each week, samples from 45 WWTPs are collected, analyzed, results reported to Health authorities, and finally published within less than 72 h in an online dashboard (https://sarsaigua.icra.cat). After 20 months of monitoring (July 20–March 22), the standardized viral load (gene copies/day) in all the WWTPs monitored fairly matched the cumulative number of COVID-19 cases along the successive pandemic waves, showing a good fit with the diagnosed cases in the served municipalities (Spearman Rho = 0.69). Here we describe the roadmap of the design and deployment of SARSAIGUA while providing several open-access tools for the management and visualization of the surveillance data.

## Introduction

Shortly after the onset of the outbreak of the current COVID-19 pandemic, scientists renewed their interest in the application of wastewater-based epidemiology (WBE)^1^ to track the communal circulation of SARS-CoV-2 through the quantification of its genetic traces in sewage^2^. This resulted in an overwhelming number of publications reporting good correlations between the load of SARS-CoV-2 entering urban wastewater treatment plants (WWTPs) and COVID-19 cases reported in the area served by these plants^3–5^. The potential capacity of WBE to anticipate surges in clinical cases (early warning) and its applicability at different spatial scales (from discrete facilities to large cities) raised the attention of public and private sectors to implement WBE of SARS-CoV-2 as a complementary tool to the epidemiological surveillance based on clinical diagnostics. In this regard, the European Commission published in March 2021 a statement encouraging Member States to put in place national wastewater surveillance systems of SARS-CoV-2 and its variants before October 2021^6^ to help Health authorities in the management of the COVID-19 pandemic.

Despite the great amount of information on the application of WBE principles to track the circulation of SARS-CoV-2 among target populations, there is a need for recommendations about the best methodological practices and technical issues regarding sample collection and concentration, RNA extraction, selection of adequate gene targets, key steps in their quantification using RT-qPCR, data interpretation, and communication of results^7^. The availability of such technical guidance provides a robust framework to scale up the analysis of SARS-CoV-2 in sewage for its application on WBE, but few insights have been published regarding the overall design of an operational surveillance network at a national scale. In this regard, the Centers for Disease Control and Prevention in the USA have published guidance for an optimal sampling strategy (*i.e.*, criteria for site selection, characteristics of the sewerage system, COVID-19 prevalence, among others)^8^ but no recommendations are provided about the nedeed steps when designing and deploying such surveillance networks. The commission recommendation advises European member states to sample WWTPs serving > 150,000 inhabitants as a minimal requirement for a nationwide surveillance sewage network^6^.

Tracking of SARS-CoV-2 in sewage was firstly implemented in The Netherlands soon after the first evidence of fecal shedding of SARS-CoV-2 was reported^2^. The Dutch sewage surveillance network started in February 2020 by monitoring seven WWTPs (6 cities and 1 airport)^5^ and gradually expanded until reaching all WWTPs in the country (352 locations) by September 2020^9^. Following the Dutch example, many countries started similar sewage surveillance programs in 2020 (e.g., Australia, Belgium, Canada, Finland, France, Italy, United Kingdom, USA, Spain, and Sweden)^10–15^. At the time of writing, 68 countries have implemented SARS-CoV-2 sewage surveillance programs at different scales and coverages as reported on the COVIDPoops19 website^16^ (https://www.covid19wbec.org/covidpoops19).

In Catalonia, an Autonomous Community of Spain, several research groups readily applied this approach to shed light on the communal circulation of SARS-CoV-2 both in the Barcelona metropolitan area during the first COVID-19 wave (March–June 2020)^17^ and in several WWTPs of different sizes during the first and second pandemic waves (March–November 2020)^18^. During this inter-wave interval, the Catalan Water Agency (ACA) and the Public Health Agency of Catalonia (ASPCAT), promoted and funded the deployment of the Catalan Surveillance Network of SARS-CoV-2 (SARSAIGUA). This network started in July 2020 monitoring 56 WWTPs evenly distributed across Catalonia and serving 80% of the total population (7,5 million inhabitants). The main goal of this paper is to describe the step-by-step process for the design and deployment of the SARSAIGUA program by including aspects related to (i) the selection criteria of sampling sites and sample collection; (ii) the optimization of shipping routes for timely sample delivery; (iii) the design of a dedicated, web platform for the collection of WWTP and laboratory metadata; (iv) the SARS-CoV-2 analysis and interpretation of results, and (v) the key steps on the design of an online dashboard for data visualization. To the best of our knowledge, this is the first paper describing the full roadmap for the implementation of a WBE surveillance network at a large scale, from its early design, deployment, and visualization of results.

## Network design

In April 2020, ICRA was commissioned by the ACA and the ASPCAT to design and implement a surveillance network to track the circulation of SARS-CoV-2 in sewage across Catalonia. The main goal was to generate quantitative data on the spread of SARS-CoV-2 in the Catalan population that could be used by Health authorities to complement the clinical epidemiological indicators in their decision-making and management of the COVID-19 pandemic. SARSAIGUA was approved by the Catalan Government on the 20th of June 2020, based on the state of alarm decreed by the Spanish authority (RD 463/2020) resulting from the health crisis caused by the pandemic. The sample collection and analysis started on the 6th of July 2020, approximately four months after the detection of the first clinic COVID-19 case in Catalonia (25th February 2020). The SARSAIGUA roadmap, from its design to its final implementation, is described in the following sections.

### Selection of WWTPs

The current number of urban WWTPs in Catalonia is 532, serving 97% of the Catalan population. The first step in SARSAIGUA was to select which WWTPs would be monitored and at which frequency, considering the maximum number of weekly samples that could be analyzed per laboratory (see “Laboratory analyses”). The selection was carried out by the coordination team, the ACA, and the ASPCAT, and it was based on two criteria: (i) to reach the maximum percentage of the assisted population, and (ii) to evenly cover the whole Catalan territory. The operational and technical capabilities of each WWTP in terms of sampling equipment (i.e., automatic samplers able to collect 24-h composite samples) and the flow data availability were also considered to refine the final selection.

**Figure 1:**
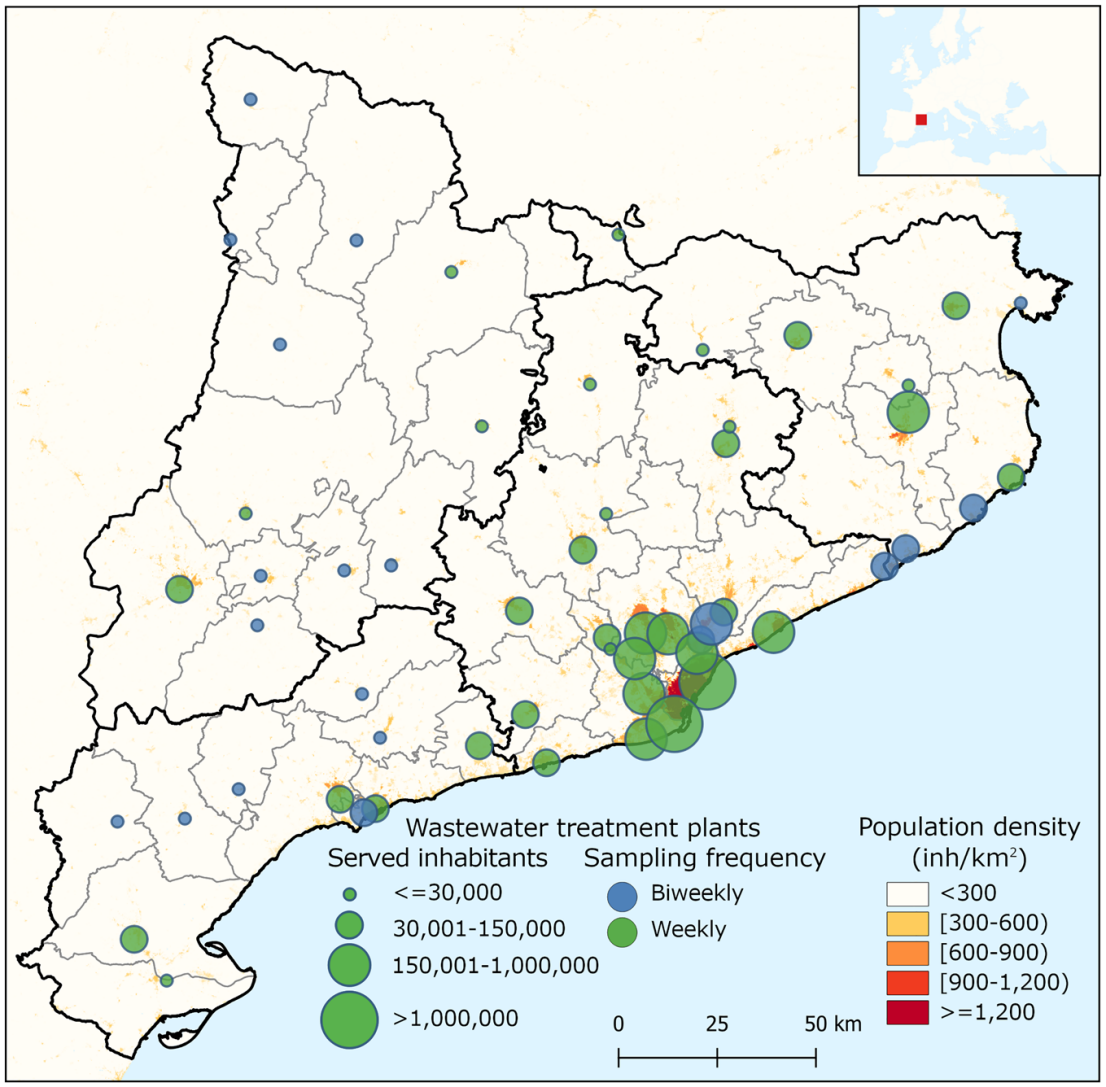
Map showing the location, the assisted population, and the sampling frequency of the 56 WWTPs finally selected within SARSAIGUA. Population density is shown using a grid with a resolution of 0.25 km^23^. Population density information was obtained from the Global Human Settlement Population Grid 2015 (European Joint Research Center, https://ghsl.jrc.ec.europa.eu/ghs_pop.php). The main map has been built using QGIS 3.22 (https://www.qgis.org/en/site/) and county limits obtained from the Cartographic and Geological Institute of Catalonia (https://www.icgc.cat/ca/Descarregues/Cartografia-vectorial/Divisions-administratives). The upper map has been obtained from the ArcGIS Hub using ESRI data (https://hub.arcgis.com/datasets/esri::world-countries-generalized).

The WWTP selection procedure was a stepwise process considering served population from each WWTP as reported by the ACA and territorial equilibrium calculated as the average distance of any point in Catalonia to the closest WWTP included in the network. Calculations were based on Euclidean distances and carried out in R software^19^ version 4.1 using packages *sp*^20^ and *raster*^21^. First, we selected those WWTPs serving more than 150,000 inhabitants thus resulting in 7 WWTPs that cover 50% of the population (threshold A in Suppl. Fig. S1). These larger WWTPs are mainly located in the Metropolitan Area of Barcelona (Fig. 1). Second, we added the largest WWTP of each Catalan County that were not included in the previous step. This selection was conducted for 41 out of the 42 Catalan counties fulfilling the technical requirements for proper sample collection (see “Sample collection and delivery”). At this second step, 42 WWTPs (including the 7 WWTPs from the first step) were selected, resulting in a 61.92% population coverage (threshold B in Suppl. Fig. S1). Third, the largest remaining WWTPs were finally added to the list until reaching a satisfactory coverage of up to 80% of the Catalan population without compromising the analytical and budgetary limits (threshold C in Suppl. Fig. S1, 56 WWTPs). As a result, we obtained an even distribution of selected WWTPs across the territory while maintaining a high population coverage (80%) (Suppl. Fig. S1). Data above threshold C in Suppl. Fig. S1 shows the cumulative incorporation of most of the remaining WWTPs in Catalonia (422 out of 532) until reaching almost total population coverage (close to 97%), a scenario that was far beyond the analytical capacity and budget of the network. Figure 1 displays the distribution of the WWTPs finally selected. Overall, the total cost (including laboratory analyses, coordination costs, web design, and sample shipment) for the monitoring of the 56 WWTPs selected was budgeted for 396,836€ for the first 6 months (July–December 2020). In December 2020, the Catalan Government extended the surveillance program until December 2022 with an additional cost of 947,430€/year^22^.

Sampling frequency was set to one sample per week in 36 of the selected 56 WWTPs and biweekly for the remaining 18, thus resulting in the collection and analysis of 45 samples per week. Remarkably, some WWTPs are only surveilled during the summer season to better monitor municipalities receiving high tourism (e.g., Castell-Platja d’Aro, Vilaseca-Salou). Details of sample collection and sampling frequency for all the monitored WWTPs are shown in Suppl. Table S1.

### Sample collection and delivery

Despite the controversy about the best strategy for the collection of wastewater samples for WBE studies (i.e., grab vs. composite samples)^24^, SARSAIGUA opted to collect flow-based composite samples at the entrance of the selected WWTPs, using refrigerated autosamplers. This strategy was adopted according to studies evidencing that composite samples offer more representative and less variable results than grab samples regarding the quantification of viral particles^24–26^ and micropollutants^27^, and also aligned with the recommendations later proposed by the European Commission^6^. Yet, several technical constraints regarding the number of available autosamplers, limitations for installing sampling equipment, and the presence of sampling points with reject water hampered the use of this approach in all the monitored WWTPs. In this regard, 91% (51 out of 56) of the monitored WWTPs carry out a flow proportional composite sampling (Suppl. Table S1). Besides, 48 out of the 56 plants (85%) collect sample fractions at 20 min intervals and the remaining 8 (15%) collect hourly fractions. Sampling is conducted from 8:00 am on Mondays to 7:40 am on Tuesdays and the composite samples are then prepared, either automatically or manually, using the available fractions. To facilitate the entire process and the sample handling during collection and storage, we did prepare a detailed sampling protocol that was distributed and explained to the WWTPs staff through online training sessions before the beginning of the first sampling campaign (6th July 2020; sampling protocol available upon request).

After collection and preparation, weekly composite samples are shipped on Tuesday morning to the laboratories in charge of the molecular analyses (see next section). To optimize time and resources, we mapped out the best shipping routes considering: (i) the starting time of delivery (Tuesday, 8:00 h); (ii) the latest delivery time to the laboratories (Tuesday, 15:00 h), and (iii) the distances and roadways between the different WWTPs. Due to the varying frequency of monitored WWTPs (weekly or biweekly) and the changing seasonal schedule for some WWTP, we finally calculated eight optimized shipping routes using package *mapsapi*^28^ in R software (see “Code availability” section).

### Laboratory analyses

The 45 weekly samples are distributed to the three reference laboratories with wide expertise in molecular diagnosis and environmental virology, two of them at the University of Barcelona, namely: the Laboratory of Enteric Viruses (https://is.gd/GFSH7W) and the Laboratory of Viruses Contaminants of Water and Food (https://is.gd/rNmGr1); and the third one in Reus, at EURECAT (https://eurecat.org/es/) composed by the Omic Sciences Unit and the Nutrition and Health Unit. All three laboratories fully comply with all necessities required for the planned analyses, including an internationally recognized expertise in environmental virology and having optimized and validated protocols to quantify viruses in complex environmental matrices. Also, all samples are handled and processed in a Biosafety Level 2 laboratory to minimize risks to the user and the environment, and to mitigate the cross-contamination of samples. Each laboratory receives 15 samples per week that are analyzed for SARS-CoV-2 genome abundance using optimized protocols. Particularly, sewage samples are concentrated using either aluminum hydroxide adsorption-precipitation concentration method^29^, ultrafiltration using either Centricon devices (Millipore Corp.)^5^, or the CP-Select concentration pipette (InnovaPrep LLC)^30^. Replicates of all water samples are kept at 4ºC for a week as a backup. Moreover, the remaining viral concentrates are frozen at –80ºC and used only in case of failure of downstream molecular analyses (see below).

Quantification of SARS-CoV-2 genomes is accomplished using RT-qPCR targeting a common genetic marker (N1) and two complementary targets, N2 and IP4^31,32^. Average values of RT-qPCR performance parameters for the three gene targets analyzed are provided in Supplementary Table S2. To control the efficiency of the entire procedure, a viral surrogate (Transmissible gastroenteritis virus (TGEV) coronavirus or MS2 bacteriophage) is added to the raw water samples as well as the processing control required to calculate the absolute recovery value per sample (see ^11^ and ^20^ for details). Additionally, considering the different analytical procedures carried out by each laboratory, several inter-laboratory comparison trials have periodically been conducted (*ca.* every three months during the first year) using raw samples, sample concentrates and synthetic RNA replicates to ensure an acceptable level of variability in results (z-score < 1, data not shown)^33^.

## Network deployment

### Data collection and curation

Basic information about sampling as well as physicochemical data from collected wastewater (e.g., time of sampling, 24-h flow, the concentration of suspended solids, phosphorous, and ammonia among others) at the inlet of the WWTP are weekly reported by plant managers using an online questionnaire created ad-hoc by the coordination team (Form 1, see “Code availability” section). Once completed, this form automatically creates a specific sample tracking code that is used for shipment and downstream analyses.

Similarly, the laboratories also enter the details of the analyzed samples and the molecular analyses using a second online questionnaire (Form 2, see “Code availability” section). In this case, this questionnaire includes details of the analytical procedure (e.g., sample volumes, volumes of final concentrates, volumes of concentrate used for RNA extraction, among others) as well as key variables of the RT-qPCR run (slope, intercept, and efficiency of the RT-qPCR reaction as well as the resulting Cq values of the samples analyzed). Besides, this form automatically computes the final concentration (in gene copies (GC)/L) of gene targets per sample. Once completed, the coordination team checks the reported values to ensure data validity and integrity before sending them to the Public Health Agency of Catalonia. After this quality control, results are published *online* through the public dashboard (https://www.sarsaigua.icra.cat) within 72 h after sample collection.

### Online public dashboard

**Figure 2:**
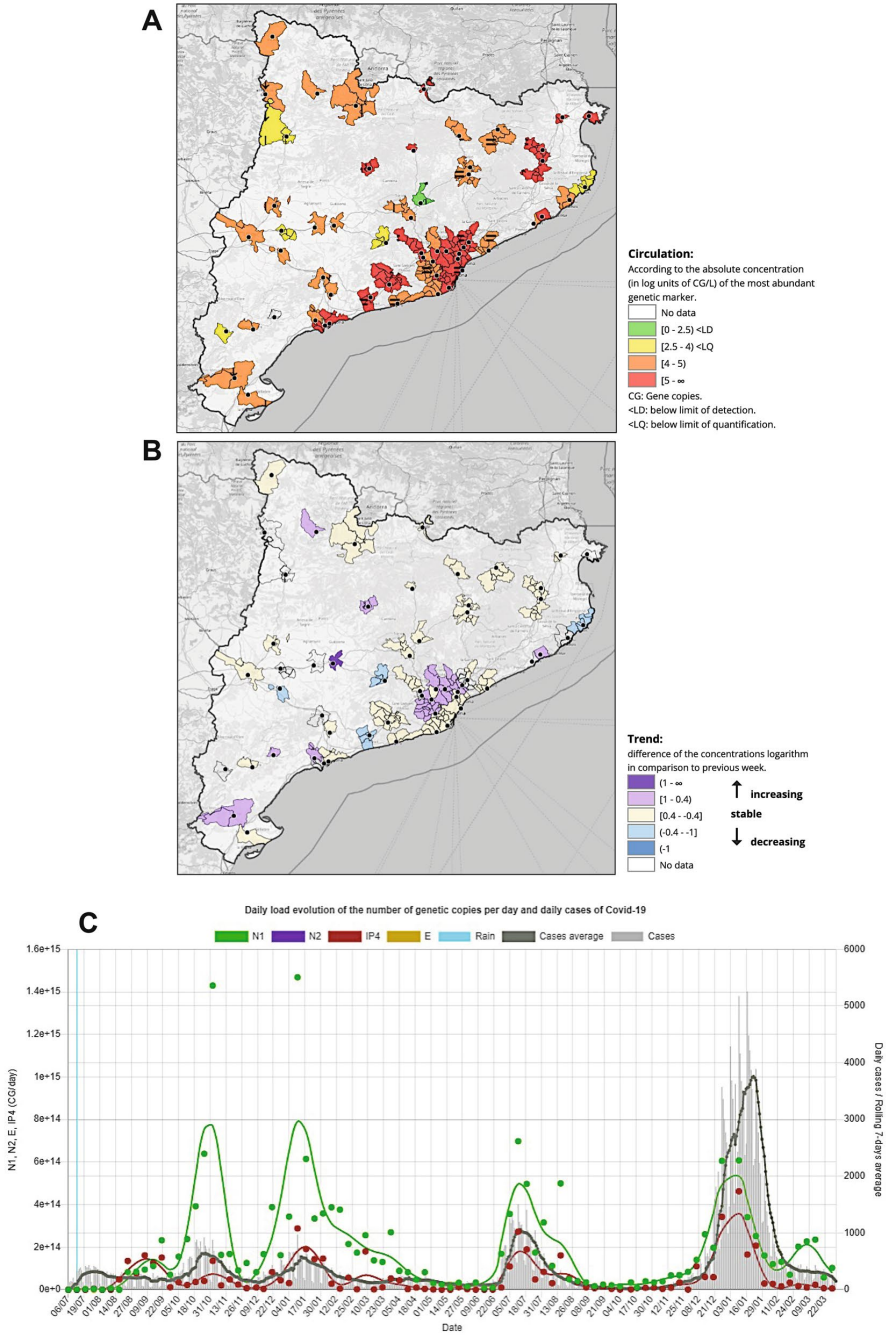
Images retrieved from the SARSAIGUA dashboard showing (**A**) the absolute abundance of SARS-COV-2 (in GC/L) at the monitored WWTP, and (**B**) weekly trends calculated at each monitored WWTP. For each WWTP, the temporal dynamics of its viral load and reported clinical cases in the assisted municipalities can also be displayed (**C**). Maps in the online dashboard were obtained from OpenStreetMap (https://www.openstreetmap.org/).

The platform facilitates public access to all results by displaying a map of Catalonia where the monitored sites (and served municipalities) are color-coded according to the absolute concentration (in log units of GC/L) of the most abundant SARS-CoV-2 genetic marker (Fig. 2A). The weekly temporal trend of SARS-CoV-2 concentrations is also displayed in an additional layer using different color codes (increasing, steady, decreasing) (Fig. 2B). The trends are calculated using the mean difference of the log-transformed concentrations of the target genes between two consecutive weeks. We set a cut-off value of ± 0.4 logs to define a significant increasing or decreasing trend in viral concentrations between consecutive weeks after assessing the analytical variability of RT-qPCR determinations by each laboratory using sample replicates (data not shown).

Details at the WWTP scale can be easily visualized by clicking the monitored sites on the map. By doing so, the temporal dynamics of the viral loads are graphically displayed in a new window (Fig. 2C). At each WWTP, viral load is calculated by multiplying the absolute concentration of targets (in GC/L) by the 24-h flow at the inlet of the WWTP. To allow a direct comparison between viral load in sewage and reported clinical cases in a given area, the latter are also displayed in the plots. Daily reported clinical cases together with their moving 7 days average were aggregated for municipalities assisted by each WWTP using an Application Programming Interface (API) created *ad-hoc* to obtain updated epidemiological information of each defined area from the Information Systems of the Department of Health and the Catalan Health Service^34^ (see “Code availability” section).

### Communication

**Figure 3:**
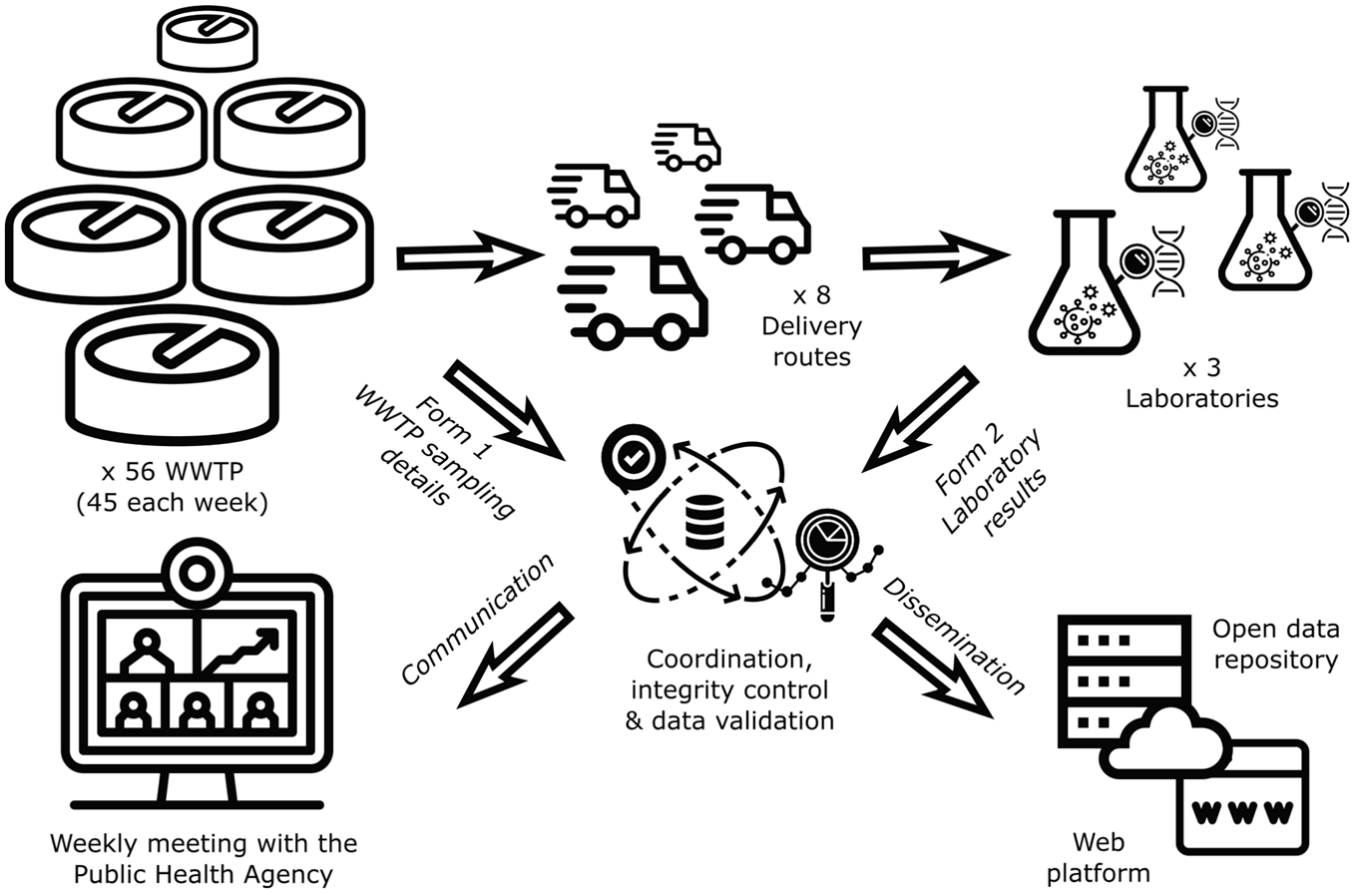
Communication pipeline of SARSAIGUA.

The communication pipeline was implemented before starting the network to ensure adequate data storage, usage, and tracking. Figure 3 illustrates the whole pipeline, from sampling collection to the online publication of results.

The communication pipeline starts as soon as the WWTPs collect the sample and upload the sampling metadata into Form 1. After sample processing and analysis, laboratories introduce results into Form 2.﻿ Once all data are validated by the coordination team, weekly results are delivered to the ASPCAT and the ACA within 72 h after sample collection. A weekly report is sent to the Catalan health authorities, namely: the General Subdirectorate for Food Safety and Health Protection of the Catalan Government, the five Regional Offices of the Public Health Agency of Catalonia, and the General Subdirectorate for Epidemiological Surveillance and Public Health Emergency Response. Authorities use this information as a complementary indicator in their decision-making process for the management of the pandemic situation in Catalonia.

After approval by Health authorities, all data are automatically uploaded both to the online public dashboard (https://sarsaigua.icra.cat) and to the public repository (see “Code availability” section) on Fridays at noon.

## Performance

After 20 months of monitoring (from July 2020 to March 2022), SARSAIGUA has analyzed approximately 3600 samples and all data generated have been uploaded in the online forms with high compliance (97.4% and 100% of completeness for forms 1 and 2, respectively). On average, results have been reported in 57.0 ± 15.8 h (2.37 ± 0.66 days) after sample collection.

**Figure 4:**
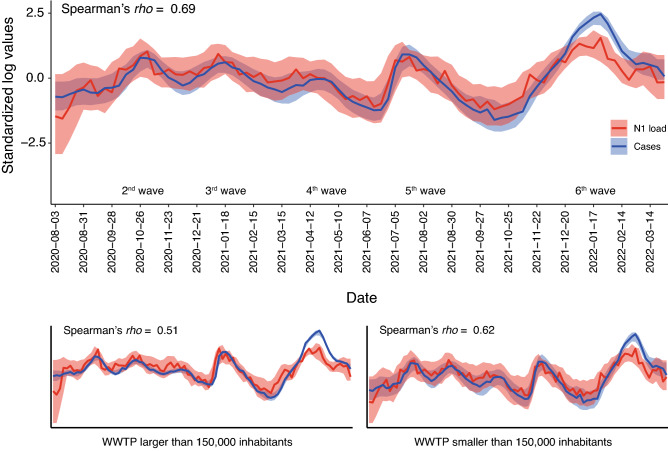
Timeline of SARS-CoV-2 standardized load in sewage (red) and diagnosed COVID-19 cases (blue) for each sampling data across all WWTP monitored. Shadowed ribbons represent the correspondent standard deviation values for each sampling date. The standard deviation values for each sampling date were smoothed using a rolling average with a window of 3 observations. The bottom plots show the same timeline when segregating viral loads and cases for large (> 150.000 inhabitants, left) and small (< 150.000 inhabitants, right) WWTPs. Values of Spearman Rho correlation coefficients are also shown. Case data have been obtained from the Information Systems of the Department of Health and the Catalan Health Service^34^.

The daily load of SARS-CoV-2 markers in the 56 WWTP monitored fairly matched the sum of COVID-19 cases along the successive pandemic waves, including the 6th wave caused by the rapid surge of Omicron cases during December 2021 (Fig. 4). To evaluate the correlation, aggregated cases of the last 7 days and N1 loads (N1 genomic copies/day) were log10 transformed and then standardized by subtracting the mean and dividing by the standard deviation. Overall, a good fit was obtained between the viral load (GC/day) and the evolution of diagnosed cases in the municipalities served by the WWTPs (Spearman correlation coefficient = 0.69). Segregation of data between large (serving > 150,000 PE) and small WWTP (< 150,000 PE) yielded a similar fit (Spearman correlation coefficient = 0.51 and 0.62 for large and small WWTPs, respectively) (see bottom panels in Fig. 4).

## Perspective and outlook

Here we describe the roadmap of the design and deployment of SARSAIGUA while providing several open-access tools created *ad-hoc* for easy data collection, storage, and calculation of results (see Supporting Material and “Code availability” section). SARSAIGUA was one of the first surveillance networks deployed in Europe and it is fully supported by public funding from the Catalan Government until December 2022 so far. SARSAIGUA benefits from the active involvement of both authorities and experts from the Health and Water sectors of the Catalan Government, which have participated in the design, implementation, and weekly monitoring since its creation. From the first design, the network fulfills 21 out of the 27 recommendations raised by the European Commission to establish systematic surveillance of SARS-CoV-2 in wastewater^6^, which is of special relevance taking into account that the program was launched 9 months before that publication (March 2021). In November 2021, SARSAIGUA built in a monthly sequencing program to track the circulation of SARS-CoV-2 variants in sewage, thus strengthening compliance with the EC recommendations.

Two key elements of the program deserve special consideration: (i) the design and functioning of the web platform that allows the rapid introduction of data, their quick validation by the coordination team, and the final visualization of results in 72 h; and (ii) the open-access policy that permeates the entire program—from the bunches of code to create the on-line formularies to the freely available, weekly updated, full dataset (see above).

However, SARSAIGUA has not been exempt from difficulties and it also suffers from some limitations. The former was mainly related to the inherent complexity of wastewater that is common to all WBE studies (see ^26^ and reference therein) and similar surveillance networks (e.g., the presence of inhibitor compounds, the mix of industrial and urban wastewater, the dilution caused by stormwater, among many others). Such complexity may lead to inconsistent results that deserve careful revision and data curation. The latter may arise for different reasons (sampling frequency, seasonal variation in the population assisted by a given WWTP, mobility of commuters, just to cite some) and erode the usefulness of the approach in providing a reliable assessment of the SARS-CoV-2 prevalence and, more important, the early detection COVID-19 outbreaks. Our sampling frequency (once a week) might restrain the anticipation capacity of the network as recently suggested^35^. Regarding the future, our efforts for improvement will be focused on increasing the sampling frequency to EC recommendations (twice a week)^6^ or even more to fully exploit the anticipation capacity of the network and to allow the estimation of SARS-CoV-2 reproductive number^36^. When clinical testing is reduced (low transmission periods) or when mild case reporting is discontinued (as of 28th March 2022 in Catalonia) sewage data becomes priceless for surveilling community transmission. The capacity to early detect transmission hot spots after a local surge in sewage viral concentrations may save costs and lives by leveraging when and where to intensify the clinical testing. Indeed, the usefulness of a sewage surveillance sentinel is particularly relevant under the current scenario where new emerging SARS-CoV-2 variants outcompete old ones on a weekly time scale.

A final consideration refers to the cost of sewage surveillance and its usefulness in the future to come. For SARSAIGUA, the annual cost of the weekly monitoring of a single WWTP is about 20,000 €. This value is small when compared to the cost of carrying out a massive screening of the population assisted by an average-size WWTP using standard diagnostic tests (i.e., antigen or PCR). Despite the uncertainties that limit the translation of the wastewater concentration of SARS-CoV-2 to individual cases, 20,000€/year/WWTP is a reasonable investment assuming the valuable information resulting from such monitoring, especially when combined with clinical epidemiological data or in scenarios when clinical testing is insufficient or unavailable. The COVID-19 pandemic has put WBE surveillance programs in the spotlight and, according to their usefulness, they are here to stay since this will surely not be the last pandemic we will have to face. As the EU commission stated, these programs will reinforce Public Health by improving resilience against health threats not contained by biological, physical, or geographical barriers^37^.

## Supplementary Information

Supplementary Information.

## Data Availability

All data is free for scientific use, and it can be downloaded from a public repository on the Zenodo website (https://doi.org/10.5281/zenodo.4147073). The database is weekly updated and contains all available physicochemical and molecular data obtained from the samples analyzed.

## Code availability

The online tools developed to design and run the *SARSAIGUA* program are provided at the following link https://covidvigilancia.icra.cat/architecture/. The key elements of the internal architecture of the platform are described in the “General diagram of the platform”. The data forms used to collect information from the WWTP and the laboratories analyzing results are described in “Form 1-WWTP” and “Form 2-LAB”. The script used to calculate the optimized routes of transport for the samples across the territory is described in “Script routes”. Finally, the API created to obtain clinical cases from served municipalities is available in “API cases”.
