# Supplementary material for: The Catalan Surveillance Network of SARS-CoV-2 in Sewage: design, implementation, and performance
Source: Sci Rep. 2022 Oct 6;12:16704. doi: 10.1038/s41598-022-20957-3 (PMC9537440; doi:10.1038/s41598-022-20957-3)
Supplement: Supplementary file 1 — Supplementary Information. [file 41598_2022_20957_MOESM1_ESM.pdf]

The Catalan Surveillance Network of SARS-CoV-2 in Sewage: design, implementation, and performance

Laura Guerrero-Latorre, Neus Collado, Nerea Abasolo, Gabriel Anzaldi, Sílvia Bofill-Mas, Albert Bosch, Lluís Bosch, Sílvia Busquets, Antoni Caimari, Núria Canela, Albert Carcereny, Carme Chacón, Pilar Ciruela, Irene Corbella, Xavier Domingo, Xavier Escoté, Yaimara Espiñeira, Eva Forés, Isabel Gandullo-Sarró, David Garcia-Pedemonte, Rosina Girones, Susana Guix, Ayalkibet Hundesa, Marta Itarte, Roger Mariné-Casadó, Anna Martínez, Sandra Martínez-Puchol, Anna Mas-Capdevila, Cristina Mejías-Molina, Marc Moliner i Rafa, Antoni Munné, Rosa Maria Pintó, Josep Pueyo, Jordi Robusté-Cartró, Marta Rusiñol, Robert Sanfeliu, Joan Teichenné, Helena Torrell, Lluís Corominas and Carles M. Borrego

Supplementary Material

Contents

1. Tables: Supplementary Table S1

2. Tables: Supplementary Table S2

3. Figures: Supplementary Figure S1

Supplementary Table S1. Details of sample collection for the 56 WWTP monitored by SARSAIGUA. BCN: Barcelona; GIR: Girona; LLE: Lleida, TAR: Tarragona.

	WWTP code	WWTP name	Province	Sampling frequency	Collection before grit chamber	24h samples	Flow proportional	20 min freq.	Refrigerated
1	DABR	Abrera	BCN	Weekly	YES	YES	NO	YES	YES
2	DAMP	Amposta	TAR	Weekly	YES	YES	YES	YES	NO
3	DBAL	Balaguer	LLE	Weekly	YES	YES	NO	YES	YES*
4	DBAY	Banyoles	GIR	Weekly	YES	YES	YES	YES	YES*
5	DBBL	Les Borges Blanques	LLE	Biweekly	NO	YES	YES	YES	YES*
6	DBER	Berga	BCN	Weekly	NO	YES	YES	YES	YES
7	DBLN	Blanes	GIR	Biweekly	NO	YES	YES	YES	YES
8	DBSS	Besòs	BCN	Weekly	NO	YES	YES	YES	YES
9	DCER	Cervera	LLE	Biweekly	YES	YES	NO	YES	YES
10	DCPA	Castell-Platja d’Aro	GIR	Biweekly (summer)	YES	YES	YES	YES	YES*
11	DFAL	Falset	TAR	Weekly (winter)	NO	YES	YES	YES	YES
12	DFIG	Figueres	GIR	Weekly	NO	YES	YES	YES	YES
13	DFON	Fondarella	LLE	Biweekly	YES	YES	YES	YES	YES
14	DGAN	Gandesa	TAR	Biweekly	YES	YES	YES	YES	YES*
15	DGIR	Girona	GIR	Weekly	YES	YES	YES	YES	YES
16	DGRA	Granollers	BCN	Weekly	YES	YES	YES	YES	YES
17	DGVC	Gavà/Viladecans	BCN	Weekly	YES	YES	YES	each 1000 m3 of the inlet flow	YES*
18	DIGU	Igualada	BCN	Weekly	YES	YES	YES	YES	YES
19	DLDM	Lloret de Mar	GIR	Biweekly	NO	YES	YES	YES	YES
20	DLLE	Lleida	LLE	Weekly	YES	YES	YES	YES	YES
21	DLLL	La Llagosta	BCN	Biweekly	YES	YES	YES	YES	YES
22	DMAN	Manlleu	BCN	Weekly	NO	YES	YES	YES	YES
23	DMAS	Manresa	BCN	Weekly	YES	YES	YES	YES	YES
24	DMAT	Mataró	BCN	Weekly	YES	YES	YES	YES	YES*
25	DMDV	Montornès del Vallès	BCN	Biweekly	YES	YES	YES	YES	YES
26	DMIR	Montcada	BCN	Weekly	YES	YES	NO	YES	YES
27	DMLN	Móra d'Ebre/ Móra La Nova	TAR	Biweekly	NO	YES	YES	YES	NO
28	DMOB	Montblanc	TAR	Biweekly	YES	YES	YES	YES	YES
29	DMOF	Montferrer	LLE	Weekly	NO	NO	NO	NO	YES
30	DMRT	Martorell	BCN	Weekly	YES	YES	YES	YES	YES*
31	DOLO	Olot	GIR	Weekly	YES	YES	YES	YES	YES
32	DPAM	Palamós	GIR	Weekly	YES	YES	YES	YES	YES*

33	DPDL	El Prat de Llobregat	BCN	Weekly	YES	YES	YES	YES	YES*
34	DPSU	El Pont de Suert	LLE	Biweekly	YES	YES	YES	YES	YES*
35	DPUI	Puigcerdà	GIR	Weekly	YES	YES	YES	YES	YES*
36	DRIP	Ripoll	GIR	Weekly	NO	YES	YES	NO	YES*
37	DRSS	Roses	GIR	Biweekly (summer)	NO	YES	YES	YES	YES
38	DRUB	Rubí	BCN	Weekly	YES	YES	YES	NO	YES
39	DRUS	Reus	TAR	Weekly	YES	YES	YES	NO	YES
40	DSFL	Sant Feliu de Llobregat	BCN	Weekly	YES	YES	YES	NO	YES
41	DSLL	Sallent/Artés	BCN	Weekly	YES	YES	YES	YES	YES
42	DSOL	Solsona	LLE	Weekly	NO	YES	YES	YES	YES*
43	DSOR	Sort	LLE	Fortnightly	YES	YES	YES	YES	YES
44	DSRS	Sabadell/Riu Sec	BCN	Weekly	YES	YES	YES	NO	YES
45	DTAR	Tarragona	TAR	Weekly	NO	YES	YES	YES	YES
46	DTOT	Tortosa-Roquetes	TAR	Weekly	NO	YES	YES	YES	YES*
47	DTRG	Tàrrega	LLE	Biweekly	YES	YES	YES	YES	YES
48	DTRP	Tremp	LLE	Biweekly	NO	YES	YES	YES	YES*
49	DTRS	Terrassa	BCN	Weekly	NO	YES	YES	YES	YES*
50	DVAL	Valls Poble	TAR	Biweekly	YES	YES	YES	YES	YES
51	DVDP	Vilafranca del Penedès	BCN	Weekly	YES	YES	YES	YES	YES
52	DVEN	Riera de la Bisbal	TAR	Weekly	YES	YES	YES	NO	NO
53	DVIC	Vic	BCN	Weekly	NO	YES	YES	YES	YES
54	DVIE	Vielha e Mijaran	LLE	Biweekly	YES	YES	YES	YES	YES*
55	DVLC	Vila-Seca/Salou	TAR	Weekly (summer)	YES	YES	YES	YES	YES*
56	DVLG	Vilanova i la Geltrú	BCN	Weekly	YES	YES	YES	YES	YES*

**The autosampler is not refrigerated but we used an alternative cooling system in this case.*

Supplementary Table S2. Details about RT-qPCR analysis performed by laboratories of SARSAIGUA.

**Experimental Design**			
Definition of experimental and control groups	Each lab analyses 15/16 samples per week including a process control in each batch		
**Sample**			
Description	24h composite wastewater sample from the inlet of a WWTP		
Processing procedure	Concentration based on precipitation (Aluminium hydroxide method) or ultrafiltration (Centricon-Millipore Corp. or CP-Select-InnovaPrep LLC))		
Sample storage conditions and duration	Samples are kept at 4º until processed within the 12h after sampling		
**Nucleic Acid Extraction**			
Procedure and/or instrumentation	Magnetic extraction/Silica columns		
Name of kit and details of any modifications	Promega Maxwell® Viral Total Nucleic Acid Purification Kit, Total RNA Purification 96-Well Kit-NORGEN, QIAamp® Viral RNA Kit-Qiagen		
Inhibition testing (C_q dilutions, spike or other)	Each sample is analyzed 1/10 dilution per duplicate to evaluate inhibition		
**RT-qPCR Target Information**	N1 (CDC, 2019)	N2 (CDC, 2019)	IP4 (Pasteur, 2020)
Sequence accession number	LC528233.1	LC528233.1	NC_004718
Amplicon length (bases)	72	67	107
**RT-qPCR Oligonucleotides**	N1	N2	IP4
Primer sequences (5'-3') F	GACCCCAAAATCAGCGAAAT	TTACAAACATTGGCCGCAAA	GGTAACTGGTATGATTTCG
Primer sequences (5'-3') R	TCTGGTTACTGCCAGTTGAATCTG	GCGCGACATTCCGAAGAA	CTGGTCAAGGTTAATATAGG
Primer sequences (5'-3') Probe	ACCCCGCATTACGTTTGGTGGACC	ACAATTTGCCCCCAGCGCTTCAG	TCATACAAACCACGCCAGG
**RT-qPCR Protocol**	N1	N2	IP4
Complete thermocycling parameters	25ºC 2 min, 50ºC 15 min, 95ºC 2 min, 45 cycles (95ºC 3 sec, 55ºC 30 sec)		55ºC 20 min, 95ºC 3 min, 50 cycles (95ºC 15 sec, 58ºC 30 sec)
Reaction volume and amount of cDNA/DNA	25µl reaction volume (5µl sample)		
Primer, (probe), Mg++ and dNTP concentrations	0,4µM primers, 0,2 µM probe, 0,8 mM Mg++, 3mM dNTPs		
Polymerase identity and concentration	Following Kit specifications		
Buffer/kit identity and manufacturer	RNA UltraSense™ One-Step Quantitative RT-PCR, Luna Universal Probe One-Step, Takara RR64A		
Manufacturer of qPCR instrument	QuantStudio 1/3 Real-Time PCR System, 7900HT ABI, Stratagene Mx3005P, BioradCFX96B		

**RT-qPCR Validation***	N1			N2		IP4
Parameters of standard curves	lab #1	lab #2	lab #3	lab #1	lab #3	lab #2
Slope	–3.38 ± 0.12	–3.44 ± 0.21	–3.43 ± 0.19	–3.48 ± 0.18	–3.63 ± 0.27	–3.52 ± 0.16
y-intercept	41.20 ± 1.11	40.55 ± 1.40	40.44 ± 0.90	40.00 ± 1.55	41.57 ± 1.37	42.18 ± 1.81
Efficiency	98.06 ± 4.83	96.21 ± 7.71	95.24 ± 7.59	93.64 ± 10.54	89.31 ± 9.83	91.67 ± 10.18
R^2	0.9887 ± 0.015	0.9876 ± 0.009	0.9928 ± 0.008	0.9832 ± 0.034	0.9915 ± 0.010	0.9902 ± 0.001
Standard controls	Twist Biosciences RNA Synthetic Controls					

* Average values of the main RT-qPCR parameters for the three gene targets analyzed. Values show the mean ± standard deviation (n = 108).

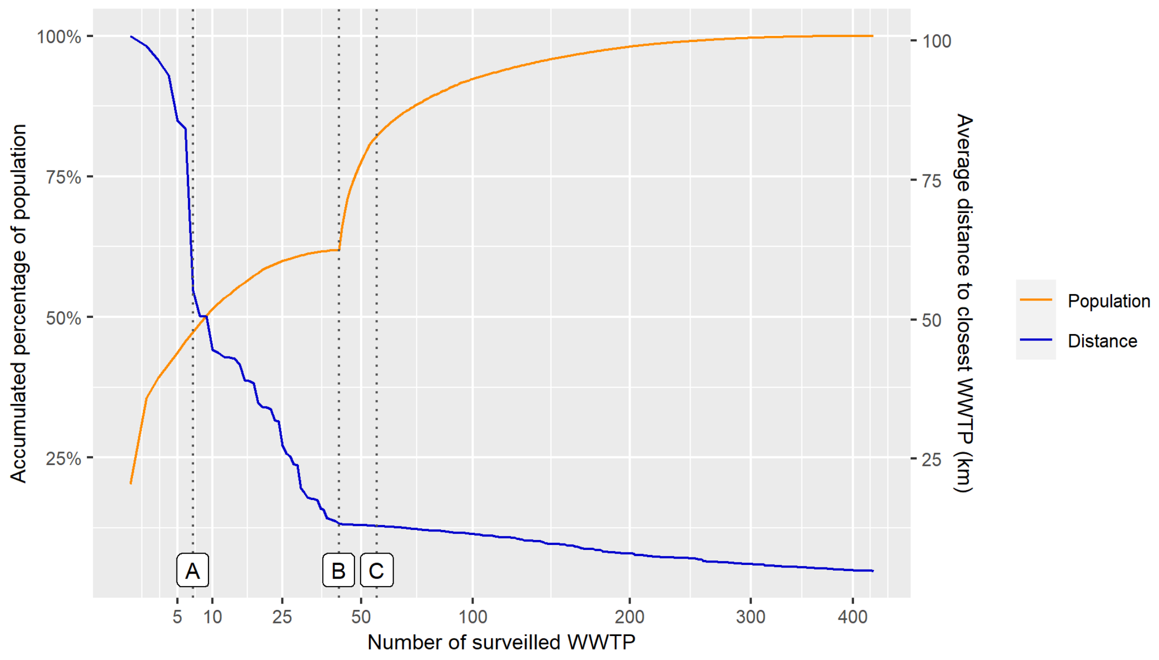

Supplementary Figure S1. Relation between surveilled WWTPs, percentage of served population and territorial equilibrium measured as the average distance of any raster cell in Catalonia to the closest WWTP included in the network. A, B and C are the thresholds in the stepwise selection procedure (A: selection of WWTPs serving > 150,000 inhabitants; B: A plus including the largest WWTPs per county; C: B plus WWTPs to reach a population coverage = 80%).
